# Timing of autologous stem cell transplantation and induction response in central nervous system lymphoma

**DOI:** 10.1007/s12185-026-04267-2

**Published:** 2026-08-19

**Authors:** Yusuke Higuchi, Hiro Tatetsu, Taichi Hirano, Takafumi Shichijo, Asami Yamada, Shikiko Ueno, Tatsuya Takezaki, Junichiro Kuroda, Naoki Shinojima, Kisato Nosaka, Akitake Mukasa, Jun-ichirou Yasunaga

**Affiliations:** 1 Department of Hematology, Rheumatology and Infectious Diseases, Kumamoto University School of Medicine, 1-1-1, Honjo, Chuo-Ku, Kumamoto, 860-8556 Japan; 2 Department of Neurosurgery, Kumamoto University Hospital, Kumamoto, Japan

**Keywords:** Primary CNS lymphoma, Secondary CNS lymphoma, ASCT, Tirabrutinib

## Abstract

Primary and secondary central nervous system lymphomas (PCNSL and SCNSL, respectively) are aggressive non-Hodgkin lymphomas with high relapse risk. Methotrexate rarely achieves durable remission, necessitating combination therapy or consolidation. Although high-dose busulfan and thiotepa (BuTT) conditioning followed by autologous stem cell transplantation (ASCT) is a promising consolidation strategy, the optimal timing and real-world outcomes of ASCT remain unclear. We retrospectively analyzed 12 patients (eight with PCNSL and four with SCNSL) treated with BuTT/ASCT at a single center between 2019 and 2024. Nine patients underwent upfront ASCT shortly after achieving complete remission with rituximab, methotrexate, procarbazine, and vincristine and remained in remission at a median follow-up of 681 days (range, 147–1322 days). Three patients (two with PCNSL and one with SCNSL) who underwent salvage ASCT following multiple prior therapies experienced early relapse (51–125 days post-ASCT) and died of their disease. BuTT/ASCT is highly effective when administered promptly after remission, whereas salvage ASCT after multiple therapies is associated with poor outcomes. Timely clinical decision-making and upfront consolidation may be key to optimizing the therapeutic benefits of ASCT in CNSL, although further validation is warranted.

**Supplementary Information:**

The online version contains supplementary material available at https://doi.org/10.1007/s12185-026-04267-2.

## Introduction

Primary central nervous system lymphoma (PCNSL) is a rare, aggressive extranodal non-Hodgkin lymphoma affecting the brain, spinal cord, leptomeninges, and ocular structures [1, 2]. High-dose methotrexate (HD-MTX) forms the backbone of initial therapy, but relapse rates remain high and long-term survival is suboptimal despite inducing responses [1, 2]. HD-MTX is frequently combined with rituximab, methotrexate, procarbazine, and vincristine (R-MPV).

Whole-brain radiotherapy may enhance local control but increases neurotoxicity risk [3–5]. Thiotepa-based high-dose chemotherapy with autologous stem cell transplantation (ASCT) has achieved favorable outcomes in newly diagnosed and relapsed PCNSL [6–8]. Most evidence derives from non-randomized phase II trials and retrospective studies, highlighting additional real-world data [2].

Secondary CNS lymphoma (SCNSL) from systemic lymphoma spread carries poor prognosis, with survival measured in months without intervention [9]. Thiotepa-based ASCT shows promise in SCNSL [10, 11].

Optimal ASCT timing, particularly whether outcomes differ between upfront ASCT after remission and salvage ASCT after multiple regimens, remains unclear. We retrospectively assessed 12 patients with CNSL who underwent busulfan and thiotepa (BuTT)/ASCT, examining how timing and treatment history affected outcomes.

## Methods

### Study design and patients

This retrospective single-center cohort study included 12 patients with PCNSL or SCNSL who underwent BuTT/ASCT at Kumamoto University Hospital between October 2019 and March 2024 (Table 1). PCNSL was defined as lymphoma confined to the CNS, whereas SCNSL was defined as CNS involvement at diagnosis or relapse of systemic lymphoma. When pathological confirmation of CNS involvement by CNS biopsy or CSF analysis was unavailable or non-diagnostic, SCNSL was diagnosed on the basis of characteristic radiological findings in the appropriate clinical context of histologically confirmed systemic DLBCL. All patients were ≥18 years of age. Histopathological confirmation of diffuse large B-cell lymphoma (DLBCL) was obtained for all cases except one. Histopathological, immunohistochemical, and molecular analyses were performed under an institutional review board-approved protocol for genetic and molecular analyses of specimens obtained from patients with brain and spinal cord tumors [12]. For one patient, cerebrospinal fluid (CSF) liquid biopsy was performed because surgical biopsy was considered high risk as the main lesion was located adjacent to the corticospinal tract and biopsy could have caused neurological deterioration. CSF samples were centrifuged, and DNA was extracted from the cellular fraction. MYD88 L265P mutation analysis was performed using digital PCR with allele-specific probes for mutant and wild-type sequences [13]. The molecular findings were integrated with radiological and clinical findings to establish the diagnosis.

**Table 1:** Patient characteristics at baseline

Characteristic	All *N*=12	Upfront ASCT *N*=9	Salvage ASCT *N*=3
*Disease, n (%)*			
PCNSL SCNSL	8 (67) 4 (33)	6 (67) 3 (33)	2 (67) 1 (33)
Age at ASCT, years, median (range)	54 (46–70)	54 (46–70)	58 (48–68)
*Gender, n (%)*			
Female Male	3 (25) 9 (75)	3 (33) 6 (67)	0 3 (100)
*ECOG PS at ASCT, n (%)*			
0 or 1 ≥2	12 (100) 0	9 (100) 0	3 (100) 0
*Previous treatment, n (%)*			
R-MPV R-ICE Tirabrutinib WBRT	12 (100) 1 (8) 2 (17) 1 (8)	9 (100) 0 0 0	3 (100) 1 (33) 2 (67) 1 (33)
*Disease status at ASCT, n (%)*			
CR/CRu PR	11 (92) 1 (8)	9 (100) 0	2 (67) 1 (33)

ASCT, autologous stem cell transplantation; CR, complete response; CRu, complete response unconfirmed; ECOG, Eastern Cooperative Oncology Group; PCNSL, primary central nervous system lymphoma; PR, partial response; PS, performance status; R-ICE, rituximab, ifosfamide, carboplatin, and etoposide; R-MPV, rituximab, methotrexate, procarbazine, and vincristine; SCNSL, secondary central nervous system lymphoma; WBRT, whole-brain radiotherapy

### Treatment

Induction therapy consisted of R-MPV. Five cycles were planned every 14–21 days. The regimen comprised rituximab 375 mg/m^2^ on day 1; HD-MTX 3.5 g/m^2^ as a 3-h infusion on day 2; vincristine 1.4 mg/m^2^ on day 2 (maximum 2 mg); and procarbazine 100 mg/m^2^/day on days 1–7 of odd-numbered cycles only. Supportive care for HD-MTX, including hydration, urine alkalinization, and leucovorin rescue, was provided according to institutional practice. Dose delays and reductions were permitted based on the patient’s clinical condition and treatment-related adverse events at the treating physician’s discretion.

**Fig. 1:**
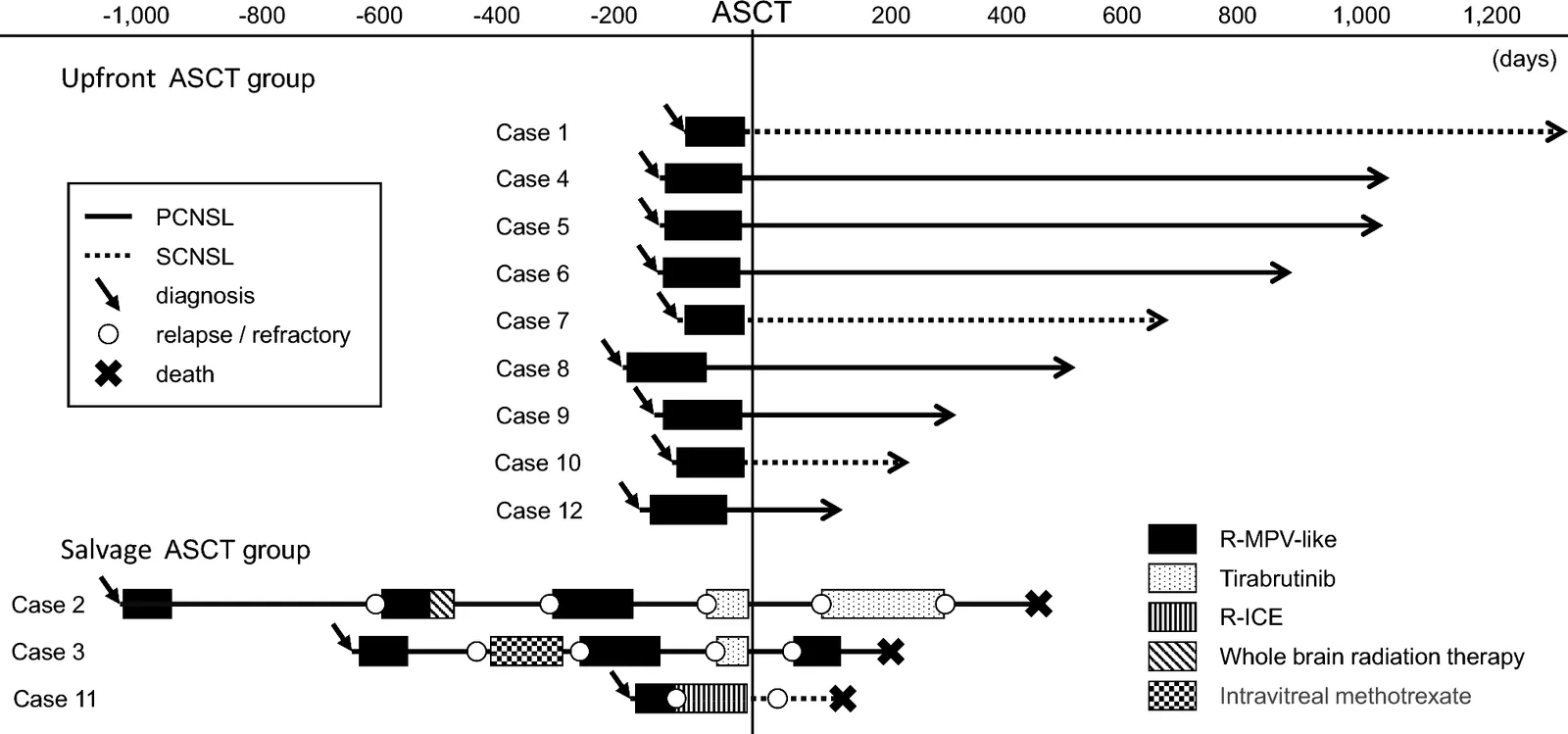
Swimmer plot demonstrating treatment timelines and relapse status for individual patients. Each swimmer plot represents a single patient and depicts the sequence and duration of treatment from diagnosis to ASCT and relapse. Different fill patterns represent various treatment modalities

Patients achieving complete remission (CR) with R-MPV were classified into the upfront ASCT group, whereas those with partial response or refractory disease received salvage therapy with tirabrutinib (a second-generation Bruton tyrosine kinase [BTK] inhibitor) or rituximab, ifosfamide, carboplatin, and etoposide (R-ICE) and were classified into the salvage ASCT group. All patients received BuTT conditioning with intravenous thiotepa (5 mg/kg on days −8 and −7) and pharmacokinetically adjusted busulfan (days −6 to −4). Peripheral blood stem cells were reinfused on day 0 with supportive care according to institutional protocol (Fig. 1)).

Treatment response in CNSL was assessed using the International PCNSL Collaborative Group criteria, whereas response in systemic lymphoma was assessed using the Lugano classification and revised International Working Group criteria for malignant lymphoma [14–16].

### Statistical analysis

Categorical variables were compared using Fisher’s exact test. Overall survival (OS) and progression-free survival (PFS) were calculated using the Kaplan–Meier method and compared using the log-rank test. OS was defined as the time from diagnosis to death or last follow-up, and PFS as the time from ASCT to relapse, progression, or death. A two-sided *p*-value <0.05 was considered statistically significant. Analyses were performed using EZR software (version 1.54; Saitama Medical Center, Jichi Medical University, Saitama, Japan) [17].

## Results

### Patient characteristics

Eight patients had PCNSL and four had SCNSL (Table 1). Median age was 54 years (range, 46–70), and nine patients (75%) were male. All patients had Eastern Cooperative Oncology Group performance status of 0–1 at ASCT. CNS involvement was predominantly parenchymal. In Case 10, MRI additionally revealed involvement of the pons, cisternal portion of the right trigeminal nerve, and Meckel’s cave, without leptomeningeal involvement. At the time of CNS involvement, extracranial systemic disease was absent in Cases 1, 7, and 10, whereas Case 11 had concurrent duodenal lymphoma at initial diagnosis. Patient-level details of systemic lymphoma treatment before CNS involvement are provided in Supplementary Table S4. Before ASCT, 11 patients achieved complete response, and one achieved partial response. Nine patients who attained complete response with first-line R-MPV comprised the upfront ASCT group. Three patients (two with PCNSL and one with SCNSL) who failed R-MPV underwent salvage therapy with tirabrutinib (*n*=2) or R-ICE (*n*=1) before salvage ASCT. Owing to the small sample size, pre-ASCT characteristics are summarized in Supplementary Tables S1 and S2, whereas treatment-related data before ASCT are summarized in Supplementary Tables S3 and S4.

### Post-transplant outcomes

**Fig. 2:**
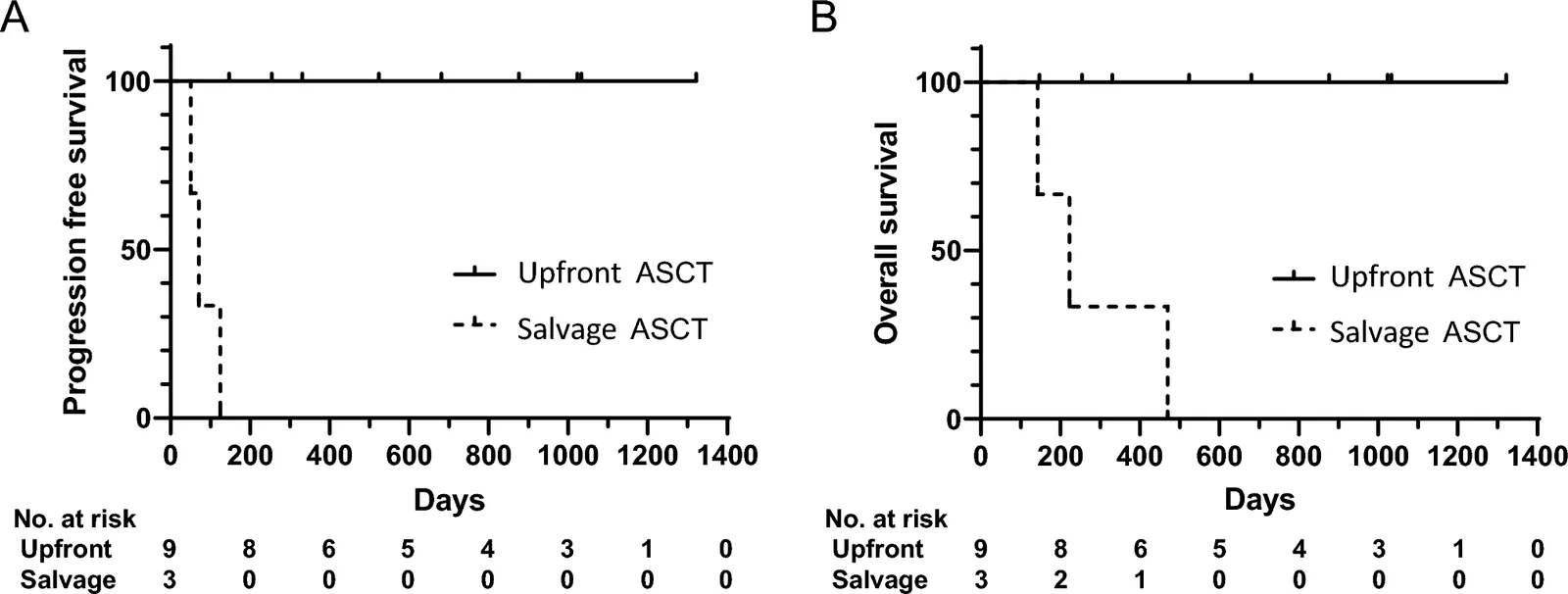
Kaplan–Meier curves demonstrating survival outcomes following busulfan and thiotepa/autologous stem cell transplantation (ASCT) in patients with central nervous system lymphoma. **A** Progression-free survival stratified by ASCT timing (upfront vs. salvage). **B** Overall survival stratified by ASCT timing (upfront vs. salvage)

All patients received BuTT conditioning and autologous stem cell reinfusion without transplant-related mortality. Hematologic recovery occurred in all patients, with neutrophil engraftment at a median of 10 days (range, 8–12). Grade ≥3 febrile neutropenia occurred in all patients (12/12), grade ≥3 hepatic toxicity in two (one grade 4 and one grade 3), and grade 3 edema in one (1/12). All other toxicities, including mucositis, diarrhea, renal toxicity, rash, and anorexia, were grade ≤2. Regarding efficacy, no transplant-related mortality occurred. In the upfront ASCT group (*n*=9), all patients remained in CR at a median follow-up of 681 days (range, 147–1322) without disease relapse or death, with 1-year PFS and OS rates of 100% (Fig. 2A and B). In contrast, all three patients in the salvage ASCT group relapsed within 51–125 days and died, with median PFS and OS of 71 and 223 days, respectively. Despite initial responses to salvage therapy, outcomes were inferior to those of upfront ASCT.

### Effect of transplant timing and treatment history

The salvage ASCT group received a median of four prior chemotherapy regimens and experienced prolonged intervals between diagnosis and transplantation. The upfront ASCT group underwent transplantation after a median of five R-MPV cycles without additional therapy (Fig. 1). The heavily pre-treated salvage cohort showed resistance and treatment failure. These findings indicate that upfront ASCT after first remission was associated with better disease control and survival, whereas salvage ASCT following multiple salvage therapies resulted in poor outcomes.

### Relapsed PCNSL treated with tirabrutinib and transplantation

**Fig. 3:**
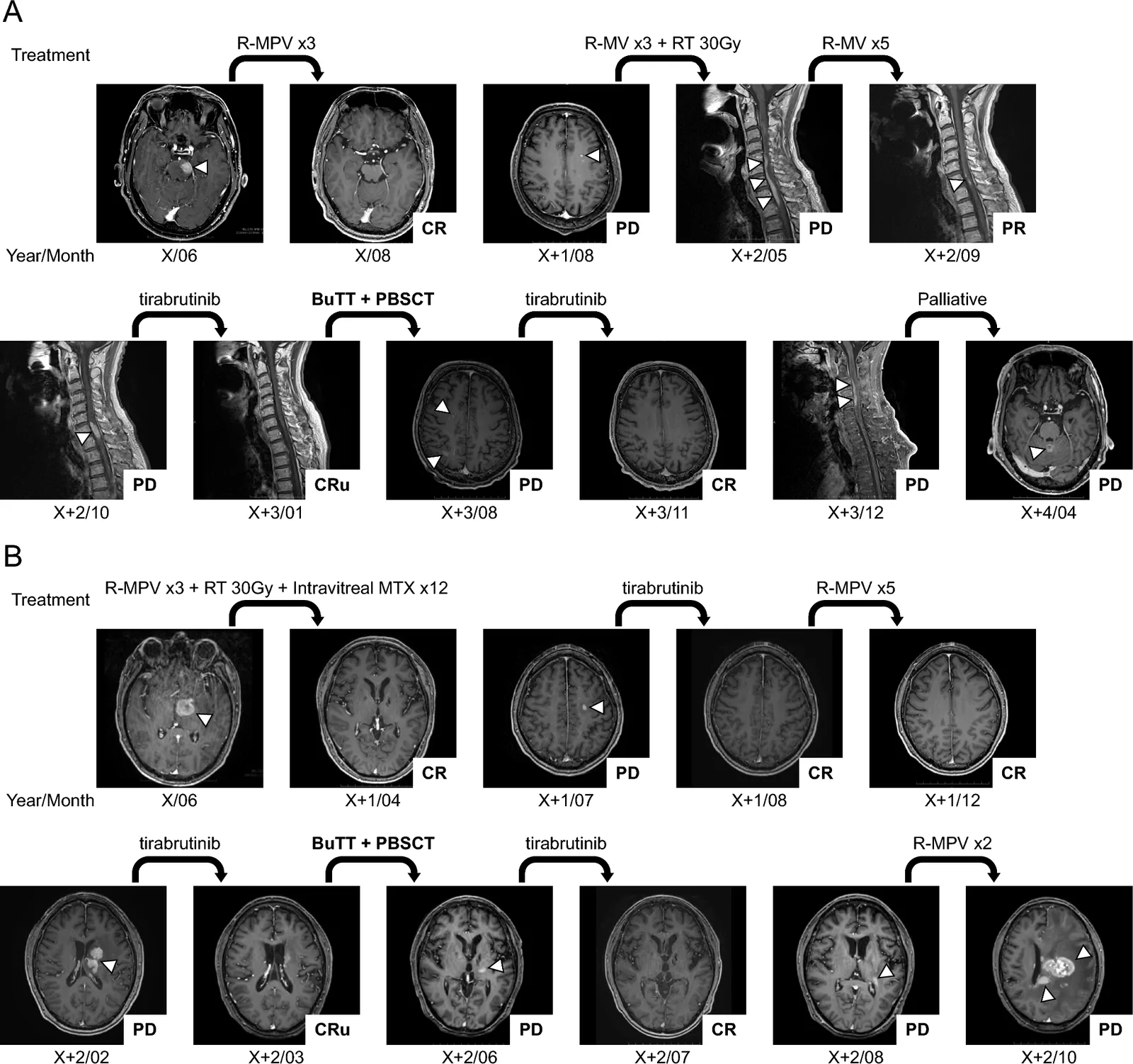
Clinical courses for two representative patients with relapsed primary central nervous system lymphoma treated with tirabrutinib before autologous stem cell transplantation (ASCT). **A** Case 2: A 59-year-old man achieved unconfirmed complete response with tirabrutinib before busulfan and thiotepa (BuTT)/ASCT but relapsed shortly after and required palliative care. **B** Case 3: A 48-year-old man achieved unconfirmed complete response with tirabrutinib before BuTT/ASCT but relapsed soon after ASCT

Two relapsed PCNSL cases highlighted an unstable, refractory course after tirabrutinib. Case 2, a 59-year-old man, achieved complete response with R-MPV, experienced multiple relapses, and attained unconfirmed complete response with tirabrutinib before BuTT/ASCT, but rapidly progressed and required tirabrutinib reintroduction and palliative care (Fig. 3A). Case 3, a 48-year-old man, responded to chemoradiotherapy and achieved unconfirmed CR with tirabrutinib after relapse, but relapsed after ASCT and progressed despite multiple salvage regimens (Fig. 3B).

## Discussion

This study highlights the importance of transplant timing in CNSL treatment. Our retrospective analysis demonstrated that those undergoing upfront transplantation after CR with R-MPV experienced better outcomes than those receiving salvage transplantation after multiple regimens. The upfront ASCT group exhibited 100% 1-year PFS and OS rates, whereas the salvage group relapsed early, with median PFS and OS of 71 and 223 days, respectively. These findings indicate the crucial role of ASCT timing in achieving favorable outcomes.

In the Japan Society for Hematopoietic Cell Transplantation (JSHCT) registry analysis of 102 patients with PCNSL who underwent HDT/ASCT between 2006 and 2015, thiotepa-containing conditioning regimens were associated with significantly superior PFS compared with non-thiotepa regimens (HR 0.42, *p*=0.038), with acceptable transplant-related mortality [18]. Consistently, OS and PFS curves favored thiotepa-based conditioning (2-year OS: 83% vs. 70%; 5-year OS: 69% vs. 52%; 2-year PFS: 72% vs. 39%; 5-year PFS: 66% vs. 32%). Because all patients in our cohort received the BuTT regimen, comparison with alternative conditioning approaches was not feasible. Nevertheless, our outcomes align with the favorable results reported for thiotepa-containing regimens. Although the JSHCT registry included upfront (*n*=65) and salvage ASCT (*n*=37), timing was not a significant determinant of outcome. However, thiotepa-specific subgroup analyses of upfront versus salvage ASCT were not performed; therefore, the effect of timing among thiotepa-treated patients remains uncertain.

Outcomes in the upfront ASCT group align with phase II trials of thiotepa-based regimens reporting high long-term remission rates when ASCT was performed during CR [6–8]. Our analysis indicates that upfront ASCT after R-MPV induction may offer similar benefits in real-world practice [19]. In contrast, poor outcomes in the salvage group highlight the risk of treatment-resistant disease because of prolonged exposure to multiple therapies and clonal evolution, consistent with studies associating relapse with increased clonal heterogeneity [20, 21]. Among the three patients who underwent salvage ASCT, inadequate response to R-MPV was a major contributing factor. Although one patient (Case 11) achieved PR with R-ICE, disease control was short-lived, suggesting that unstable chemosensitivity combined with salvage transplantation contributed to the unfavorable outcome. These findings highlight the interplay between early chemosensitivity and transplant timing. Patients who achieve durable remission after R-MPV appear to benefit most from prompt ASCT consolidation. Our favorable outcomes with upfront transplantation suggest that disease status and chemosensitivity at transplantation are critical determinants of outcome, even with thiotepa-based conditioning. This interpretation is supported by the prospective IELSG-42 (MARIETTA) trial, in which patients with newly diagnosed SCNSL had markedly better outcomes than those with CNS relapse (2-year PFS: 71% vs. 28%) [11]. These findings indicate substantial prognostic differences between newly diagnosed and relapsed CNS disease and suggest that although thiotepa-based conditioning offers a consistent therapeutic advantage, its maximal benefit may depend on performing ASCT under favorable disease conditions.

In our cohort, no transplant-related mortality occurred, and hematologic recovery was prompt, with neutrophil engraftment at a median of 10 days. These findings are consistent with a Japanese expanded-access program evaluating thiotepa (DSP-1958) plus busulfan in 51 patients with malignant lymphoma, including 25 with PCNSL. Neutrophil engraftment was achieved in 92% of patients at a median of 11 days, the 100-day survival rate was 100%, and no unexpected toxicities were reported [22]. Common adverse events included manageable febrile neutropenia, diarrhea, and mucositis, supporting the tolerability of thiotepa-based conditioning.

All three patients with salvage transplantation responded to salvage regimens, achieving partial or unconfirmed complete responses, but relapsed early after ASCT, highlighting the limitations of salvage strategies and a narrowing therapeutic window. Two patients achieved unconfirmed complete responses with the BTK inhibitor tirabrutinib before transplantation but relapsed shortly thereafter, indicating different antitumor mechanisms and resistance profiles of BTK inhibitors and high-dose chemotherapy. Sensitivity to one may not predict responsiveness to the other. This discordance highlights the need to clarify clonal dynamics and assess combinations or sequential use of novel agents. Emerging immunotherapies, including chimeric antigen receptor T-cell therapy and immune checkpoint inhibitors, warrant further investigation in this setting [23–26].

This study had some limitations. First, it was a retrospective, single-center analysis with a very small sample size, limiting statistical power and adjustment for confounders. Second, PCNSL and SCNSL are biologically and clinically distinct entities, yet both were included. The findings should not be interpreted as establishing a unified “optimal timing” strategy for all CNSL cases. In particular, SCNSL generally requires adequate extracranial disease control, and the SCNSL cases in our cohort may not represent the broader SCNSL population. Third, although pre-ASCT characteristics are provided in Supplementary Tables S1 and S2, the very small sample size limited incorporation of these clinicopathologic variables into the main analysis and disease-specific interpretation, hindered comparison with prior studies, and may have resulted in residual confounding. Finally, comparison between upfront and salvage transplantation is intrinsically confounded by treatment history, chemosensitivity, and patient selection. Patients who underwent upfront ASCT after achieving CR with R-MPV likely represented a biologically favorable, treatment-tolerant subgroup; therefore, the excellent outcomes observed may reflect the consolidative effect of BuTT/ASCT and the selection of patients with chemosensitive disease. Accordingly, our observations should be considered descriptive and hypothesis generating, warranting larger multicenter studies for refining patient selection and clarifying the role and timing of thiotepa-based ASCT in distinct CNSL subgroups.

In conclusion, upfront BuTT/ASCT after CR with HD-MTX-containing induction therapy was associated with durable disease control in patients with CNSL, whereas salvage ASCT after multiple prior therapies was followed by early relapse. These findings suggest that thiotepa-based ASCT may provide the greatest benefit when performed under favorable disease conditions and warrant validation in larger multicenter studies.

## Supplementary Information

Below is the link to the electronic supplementary material.

Supplementary file1 (DOCX 29 kb)

## Data Availability

Inquiries regarding the data should be directed to tatetsu@kumamoto-u.ac.jp. The data will be made available to achieve the aims of the approved proposal.
