# Supplementary material for: Timing of autologous stem cell transplantation and induction response in central nervous system lymphoma
Source: Int J Hematol. 2026 Aug 19;124(4):495–501. doi: 10.1007/s12185-026-04267-2 (PMC13638692; doi:10.1007/s12185-026-04267-2)
Supplement: Supplementary file 1 [file 12185_2026_4267_MOESM1_ESM.docx]

**Table S1. PCNSL-specific baseline characteristics and changes in KPS during treatment**

| **Case** | **LDH (U/L)** | **MSKCC prognostic class** | **Tumor burden (SPD, mm²)** | **CNS involvement (site)** | **CNS diagnosis** | **Cell of origin** | **KPS** | **KPS after induction therapy** | **KPS before ASCT** | **Most recent KPS** |
| --- | --- | --- | --- | --- | --- | --- | --- | --- | --- | --- |
| 2 | 149 | 2 | 977.4794 | Lt. FL, Bilat. basal ganglia/thalamus, brainstem/cerebellum | Needle biopsy | GCB | 70 | 90 | 70 | 0 |
| 3 | 214 | 1 | 902.9484 | Lt. basal ganglia/thalamus | Needle biopsy | non-GCB | 60 | 80 | 70 | 0 |
| 4 | 179 | 1 | 201.075 | Rt. FL, Rt. basal ganglia/thalamus, Lt. FL | CSF liquid biopsy (MYD88) | N/A* | 90 | 90 | 90 | 100 |
| 5 | 166 | 2 | 487.7775 | brainstem/cerebellum | Needle biopsy | GCB | 80 | 100 | 100 | 100 |
| 6 | 162 | 2 | 1264.6752 | Lt. basal ganglia/thalamus | Needle biopsy | non-GCB | 70 | 70 | 70 | 70 |
| 8 | 200 | 3 | 1257.4835 | Rt. basal ganglia/thalamus, Rt. TL | Needle biopsy | non-GCB | 20 | 70 | 70 | 90 |
| 9 | 154 | 2 | 5097.873 | Lt. FL, corpus callosum | Open biopsy | GCB | 70 | 70 | 70 | 70 |
| 12 | 244 | 3 | 2913.2763 | Rt. TL | Partial resection | GCB | 30 | 100 | 100 | 100 |

Abbreviations: PCNSL, primary CNS lymphoma; LDH, lactate dehydrogenase; MSKCC, Memorial Sloan Kettering Cancer Center; SPD, sum of the product of diameters; CNS, central nervous system; CSF, cerebrospinal fluid; Lt., left; Rt., right; Bilat., bilateral; FL, frontal lobe; TL, temporal lobe; KPS, Karnofsky Performance Status; GCB, germinal center B-cell; N/A, not assessable. *Cell of origin was not assessable for Case 4 because the diagnosis was established by detecting the MYD88 L265P mutation in a cerebrospinal fluid (CSF) liquid-biopsy specimen.

**Table S2. SCNSL-specific baseline characteristics**

| **Case** | **Primary tumor site** | **Cell of origin (primary systemic lesion)** | **CNS involvement (site)** | **CNS diagnosis** | **Brain biopsy** | **Cell of origin (CNS lesion)** | **Extracranial systemic disease at CNS involvement** | **Timing of CNS involvement (initial diagnosis vs. relapse)** |
| --- | --- | --- | --- | --- | --- | --- | --- | --- |
| 1 | liver | non-GCB | Lt. cerebellum | MRI alone (no CSF examination) | Not done | N/A | None | First relapse |
| 7 | testis | N/A | Lt. corpus callosum | MRI alone (no CSF examination) | Not done | N/A | None | First relapse |
| 10 | nasopharynx and oropharynx | non-GCB | Pons, cisternal portion of the right trigeminal nerve, and Meckel’s cave | MRI; CSF cytology (negative) | Not done | N/A | None | First relapse |
| 11 | duodenum | non-GCB | FL | Needle biopsy | DLBCL | non-GCB | Duodenal lymphoma | At initial diagnosis |

Abbreviations: SCNSL, secondary CNS lymphoma; CNS, central nervous system; DLBCL, diffuse large B-cell lymphoma; Lt., left; Rt., right; FL, frontal lobe; GCB, germinal center B-cell; N/A, not available.

**Table S3. Details of R-MPV/R-MPV-like therapy and response assessment before ASCT in patients with PCNSL**

| **Case** | **Number of R-MPV/R-MPV-like cycles** | **Dose modifications during R-MPV/R-MPV-like therapy** | **Treatment delays during R-MPV/R-MPV-like therapy** | **Interval between completion of the final pre-ASCT therapy and ASCT** | **Interval between response assessment and ASCT** | **Treatment response of the final pre-ASCT therapy** | **Treatment response of ASCT** |
| --- | --- | --- | --- | --- | --- | --- | --- |
| 2 | 11* | Procarbazine was discontinued after the first relapse because of suspected drug-induced rash | None | 1 day (tirabrutinib) | 22 days | CRu | PD |
| 3 | 8# | None | None | 4 days (tirabrutinib) | 24 days | CRu | PD |
| 4 | 5 | None | None | 43 days | 12 days | CR | CR |
| 5 | 5 | None | None | 34 days | 31 days | CR | CR |
| 6 | 5 | None | None | 27 days | 3 days | CR | CR |
| 8 | 5 | None | None | 21 days | 8 days | CR | CR |
| 9 | 5 | None | None | 48 days | 4 days | CR | CR |
| 12 | 5 | Vincristine and procarbazine were discontinued from the second cycle onward because of suspected drug-induced cytopenia and peripheral neuropathy. | None | 50 days | 14 days | CR | CR |

Treatment delay was defined as a postponement of ≥10 days from the scheduled treatment date owing to adverse events or other reasons, excluding delays related to stem cell collection.

Abbreviations: ASCT, autologous stem cell transplantation; CR, complete response; CRu, complete response unconfirmed; PD, progressive disease; PCNSL, primary central nervous system lymphoma; R-MPV, rituximab, methotrexate, procarbazine, and vincristine. *The detailed treatment course for Case 2 is shown in Figure 3A. ^#^The detailed treatment course for Case 3 is shown in Figure 3B.

**Table S4. Details of systemic lymphoma treatment, CNS-directed therapy, and response assessment before ASCT in patients with SCNSL**

| **Case** | **Pre-CNS systemic regimen and cycles** | **Timing relative to CNS disease** | **CNS-directed regimen and cycles** | **Additional salvage regimen and cycles** | **Interval between completion of the final pre-ASCT therapy and ASCT (days)** | **Interval between response assessment and ASCT (days)** | **Treatment response of the final pre-ASCT therapy** | **Treatment response of ASCT** |
| --- | --- | --- | --- | --- | --- | --- | --- | --- |
| 1 | R-CHOP ×8 | Completed before CNS relapse | R-MPV ×5 | None | 23 | 17 | CR | CR |
| 7 | R-CHOP ×8 | Completed before CNS relapse | R-MPV ×5 | None | 21 | 5 | CR | CR |
| 10 | R-CHOP ×8 | Completed before CNS relapse | R-MPV ×7 | None | 13 | 14 | CR | CR |
| 11 | None | N/A (CNS involvement at initial diagnosis) | R-MPV ×4 | R-ICE ×4 | 33 | 21 | PR | PR |

Abbreviations: ASCT, autologous stem cell transplantation; CR, complete response; PR, partial response; SCNSL, secondary CNS lymphoma; R-ICE, rituximab, ifosfamide, carboplatin, and etoposide; R-CHOP, rituximab, cyclophosphamide, doxorubicin, vincristine, and prednisone; R-MPV, rituximab, methotrexate, procarbazine, and vincristine.
